# Long-term variation in skeletal muscle and adiposity in patients undergoing esophagectomy

**DOI:** 10.1093/dote/doab016

**Published:** 2021-04-05

**Authors:** Piers R Boshier, Fredrik Klevebro, Wesley Jenq, Francesco Puccetti, Keerthini Muthuswamy, George B Hanna, Donald E Low

**Affiliations:** Department of Surgery and Cancer, Imperial College London, London, UK; Department of Thoracic Surgery and Thoracic Oncology, Virginia Mason Medical Center, Seattle, WA, USA; Department of Thoracic Surgery and Thoracic Oncology, Virginia Mason Medical Center, Seattle, WA, USA; Department of Thoracic Surgery and Thoracic Oncology, Virginia Mason Medical Center, Seattle, WA, USA; Department of Thoracic Surgery and Thoracic Oncology, Virginia Mason Medical Center, Seattle, WA, USA; Department of Surgery and Cancer, Imperial College London, London, UK; Department of Surgery and Cancer, Imperial College London, London, UK; Department of Thoracic Surgery and Thoracic Oncology, Virginia Mason Medical Center, Seattle, WA, USA

**Keywords:** esophagectomy, body composition, skeletal muscle, adiposity, sarcopenia

## Abstract

This study seeks to define long-term variation in body composition in patients undergoing esophagectomy for cancer and to associate those changes with survival. Assessment of skeletal muscle, visceral (VAT) and subcutaneous adipose tissue (SAT) was performed using computed tomography (CT) images routinely acquired: at diagnosis; after neoadjuvant therapy, and; >6 months after esophagectomy. In cases where multiple CT scans were performed >6 months after surgery, all available images were assessed. Ninty-seven patients met inclusion criteria with a median of 2 (range 1–10) postoperative CT images acquired between 0.5 and 9.7 years after surgery. Following surgical treatment of esophageal cancer, patients lost on average 13.3% of their skeletal muscle, 64.5% of their VAT and 44.2% of their SAT. Sarcopenia at diagnosis was not associated with worse overall survival (66.3% vs. 68.5%; *P* = 0.331). Sarcopenia 1 year after esophagectomy was however associated with lower 5-year overall survival (53.8% vs. 87.5%; *P* = 0.019). Survival was lower in those patients who had >10% decrease in skeletal muscle index (SMI; 33.3% vs. 72.1%; *P* = 0.003) and >40% decrease in SAT 1 year after surgery (40.4% vs. 67.4%; *P* = 0.015). On multivariate analysis, a decline in SMI 1 year after surgery was predictive of worse survival (HR 0.38, 95%CI 0.20–0.73; *P* = 0.004). This study provides new insight relating to long-term variation in body composition in patients undergoing esophagectomy for cancer. Findings provide further evidence of the importance of body composition, in particular depletion of skeletal muscle, in predicting survival following esophagectomy.

## INTRODUCTION

Esophageal cancer is a pernicious disease, associated with poor survival. At the time of diagnosis, the majority of patients are found to have advanced disease. The metabolic and physiological burden of the disease and its treatment further contribute to high rates morbidity and mortality.

The inability to meet the body’s metabolic requirements and the resultant breakdown of energy stores within muscle and fat is a common feature of esophageal cancer that is increasingly being recognized as major driver of worse clinical outcomes.^1–3^ Previous studies have reported a net depletion of weight, body mass index skeletal muscle and adipose tissue following potentially curative treatment for esophageal cancer.^2^^,^^4^^,^^5^ Recent research has sought to draw associations between parameters of body composition and treatment outcomes for esophageal cancer. This approach is appealing as it utilizes data readily available from computed tomography (CT) images acquired as part of patients routine care. Analysis of these images can provide objective measurements of skeletal muscle and adipose tissues that may serve as an adjunct to existing methods of nutritional assessment and prognostication. In previous studies preoperative sarcopenia, a state of severe muscle loss and function was shown to affect perioperative morbidity and long-term survival in patients undergoing esophagectomy for esophageal cancer.^6^ By defining variation in body composition, it may therefore be possible to gain further insight into the effects of esophageal cancer and its treatment. Such knowledge may help to improve understanding of how sarcopenia and other measures influence clinically relevant outcomes. Ultimately, this approach could be utilized to identify ‘at risk’ patients and to track their response to interventions that are intended to mitigate further deterioration in protective characteristics of body composition.

It is hypothesized that esophageal cancer and its treatment will lead to a significant and sustained decline in parameters of body composition, the magnitude of which will be predicted by patient and tumor specific characteristics. Observed changes are also expected to influence long-term outcomes, including survival. The aim of this study was therefore to define long-term variation in parameters of body composition in patients undergoing surgical management of esophageal cancer and to associate those changes with clinically relevant patient characteristics and outcomes.

## Methods

Patients who had undergone esophagectomy for esophageal cancer at Virginia Mason Medical Centre (Seattle, USA) and St Mary’s Hospital (London, UK) between January 2006 and December 2017 were assessed for eligibility. Inclusion criteria were patients with: biopsy confirmed esophageal carcinoma (adenocarcinoma or squamous cell carcinoma); elective esophagectomy; with or without neoadjuvant therapy, and; availability of both preoperative and at least one postoperative (>6 months) CT scan. Patients who underwent esophagectomy as an emergency or for an indication other than carcinoma and patients with CT images that were not of suitable quality for assessment of body composition (e.g. image deterioration from artifacts) were excluded. This study was granted local Institutional Review Board approval.

### Patient care pathway and surgical technique

Following diagnosis, all patients were discussed within a multidisciplinary tumor board which provided treatment recommendations in accordance with clinical stage and patient characteristics. Esophagectomy was performed by two surgeons (DL, Virginia Mason and GH, St Mary’s Hospital). Esophagectomy was most commonly performed by either two stage (Ivor Lewis) or left thoracoabdominal approach with radical lymphadenectomy. Ninety-five percent of procedures were via an open approach. In all patients, esophageal reconstruction was achieved through the creation of a tubularized gastric conduit of width 2.5–5 cm.

CT imaging was acquired for the purpose of clinical staging in all patients at the time of diagnosis and after neoadjuvant chemotherapy or chemoradiotherapy (where given). Following hospital discharge, patients at St Mary’s hospital were intended to undergo yearly surveillance CT imaging for a minimum of 5 years whilst at Virginia Mason surveillance CT imaging was acquired at 1 year after surgery. Additional cross-sectional imaging outside routine surveillance was acquired on a patient-by-patient basis in accordance with clinical suspicion of disease recurrence and/or other concerning pathology.

### CT assessment of body composition

Body composition assessment was performed through the analysis of contrast enhanced CT images routinely acquired: at diagnosis; after neoadjuvant therapy, and; >6 months after surgery. In cases where multiple CT scans were performed >6 months after surgery, all were acquired. From each CT, a single axial image was selected from the midpoint of the third lumbar (L3) vertebrae and saved as an anonymized Digital Imaging and Communications in Medicine file. Cross-sectional area and radiodensity of skeletal muscle (−29 to +150 HU), visceral (−150 to −50 HU) and subcutaneous (−190 to −30 HU) adipose tissues was assessed using Slice-O-Matic (version 5.0, Tomovision, Magog, Canada) and the ABACS-L3 module (version 1.0, Voronoi Health Analytics, Canada). Segmented CT images were subsequently reviewed and manually corrected by a single trained assessor (PB), who was blinded to the patient identity and image chronology. Skeletal muscle index (SMI) was calculated as the ratio of lumbar skeletal muscle area to height and was defined as SMI < 52.4 cm^2^/m^2^ for men and <38.5 cm^2^/m^2^ for women.^7^ Visceral obesity was defined as a visceral fat area > 163.8 cm^2^ for men and >80.1 cm^2^ for women. Sarcopenic obesity was defined as sarcopenia in the presence of BMI ≥ 30 kg/m^2^.^8^

### Outcome measures

The primary outcome measure of this study was to define longitudinal variation in parameters of body composition (skeletal muscle, visceral and subcutaneous adipose tissue [SAT]) in patients undergoing potentially curative treatment for esophageal cancer. Secondary outcomes were to: (i) define factors predictive of variation in body composition, and (ii) determine in the relationship between variation in body composition and long-term survival.

### Statistical analysis

Statistical analysis was performed using SPSS (version 25.0, IBM Corp., Armonk, USA). Unless otherwise stated continuous variables are presented as mean ± standard deviation. Pairwise comparison of continuous data was performed using either an independent sample *T*-test or Mann–Whitney *U* test. One-way ANOVA was used to compare longitudinal variation in continuous variables. Categorical variables were compared using the chi-squared or Fisher’s exact tests. Survival was defined by the time interval from the date of esophagectomy to the date of either death or censoring. Survival analysis was conducted through creation of Kaplan–Meier plots with statistical comparisons made using the Log Ranks test. Multivariable analysis of predictors of survival was performed by inputting variables that had univariate statistical significance *P* ≤ 0.250 or specific clinical relevance into Cox proportional hazards regression models with stepwise entry of variables into the model. Statistical significance was assigned to two-sided *P*-values < 0.05.

## RESULTS

### Patient characteristics

During the study period, 361 patients underwent esophagectomy in both institutions, of which 97 met inclusion criteria. Baseline patient characteristics are presented in Table 1. Of those patients included in this study, the majority were male (76.3%) and the average age was 63.2 ± 10.8 yrs. Adenocarcinoma was the predominant tumor subtype in both males and females, however, a significantly greater proportion of females had squamous cell carcinomas (94.6% vs. 52.2%; *P* < 0.001). Eighty percent of patients received neoadjuvant therapy.

**Table 1:** Patient characteristics

	All *N* = 97		Male *N* = 74		Female *N* = 23		*P*
Age (years)	63.2 ± 10.8		63.6 ± 10.1		62.1 ± 12.8		0.568
BMI at diagnosis (kg/m^2^)	26.7 ± 4.1		27.1 ± 3.7		25.1 ± 5.0		0.518
Dysphagia at diagnosis	68 (70.1)		53 (71.6)		15 (65.2)		0.558
Weight loss at diagnosis (kg)	7.4 ± 5.0		6.2 ± 3.4		7.9 ± 5.3		0.306
Charlson comorbidity index^a^	5 (4–5)		4 (3–5)		5 (4–6)		0.131
ECOG							
0	70 (72.2)		54 (73.0)		16 (69.6)		0.750
1	27 (27.8)		20 (27.0)		7 (30.4)		
ASA							
II	53 (54.6)		36 (48.6)		17 (73.9)		0.034
III	44 (45.4)		38 (51.4)		6 (26.1)		
Adenocarcinoma	82 (84.5)		70 (94.6)		12 (52.2)		<0.001
Squamous cell carcinoma	15 (15.5)		4 (5.4)		11 (47.8)		
Tumor location							
Upper esophagus	2 (2.1)		0 (0.0)		2 (8.7)		0.033
Lower esophagus	32 (33.0)		24 (32.4)		8 (34.8)		
GOJ	63 (64.9)		50 (67.6)		13 (56.5)		
Neoadjuvant therapy							
Chemotherapy	52 (53.6)		40 (54.1)		12 (52.2)		0.890
Chemoradiotherapy	26 (26.8)		19 (25.7)		7 (30.4)		
Stage							
	Clinical	Pathological	Clinical	Pathological	Clinical	Pathological	
I	15 (15.5)	34 (35.1)	11 (14.9)	24 (32.4)	4 (17.4)	10 (43.5)	0.885^c^
II	9 (9.3)	13 (13.4)	7 (9.5)	10 (13.5)	2 (13.0)	3 (13.0)	0.230^d^
III	62 (63.9)	37 (38.1)	47 (63.5)	32 (43.2)	15 (65.3)	5 (21.7)	
IV	8 (8.2)	13 (13.4)	7 (9.4)	8 (10.8)	1 (4.3)	5 (21.7)	
Surgery							
Open	92 (94.8)		71 (95.9)		21 (91.3)		0.589
Hybrid	5 (5.2)		3 (4.1)		2 (8.7)		
Postoperative complications							
Clavien-Dindo grade ≥ III	27 (27.8)		19 (25.7)		8 (34.8)		0.395
Anastomotic leak	4 (4.1)		3 (4.1)		1 (4.3)		1.000
Pneumonia	37 (38.1)		31 (41.9)		6 (26.1)		0.173
Respiratory failure	6 (6.2)		5 (6.8)		1 (4.3)		1.000
Myocardial infarction	1 (1.0)		1 (1.4)		0 (0.0)		1.000
PE	2 (2.1)		2 (2.7)		0 (0.0)		1.000
Sepsis	1 (1.0)		0 (0.0)		1 (4.3)		0.237
Blood transfusion	16 (16.5)		9 (12.2)		7 (30.4)		0.039
Return to theater	3 (3.1)		2 (2.7)		1 (4.3)		0.560
Length of hospital stay (days)^b^	13 (10–20)		13 (10–19)		12 (10–26)		0.575
Hospital readmission < 30 days	3 (3.1)		2 (2.7)		1 (4.3)		0.560

BMI, body mass index. ECOG, Eastern Cooperative Oncology Group performance status. ASA, American Society of Anesthesiologists score. DVT, deep venous thrombosis. PE, pulmonary embolism.

a Age adjusted Charlson comorbidity index presented as median (interquartile range).

b Length of hospital stage presents as median (interquartile range).

c Comparison of clinical stage.

d Comparison of pathological stage.

### Longitudinal variation in parameters of body composition

A total of 401 suitable CT images were acquired and analyzed. No patients were excluded from this study due to poor CT image quality. CT images were available for all patients at diagnosis (*n* = 97). Of the 78 patients who underwent neoadjuvant therapy, 61 (78.2%) had restaging CT imaging available prior surgery. Postoperative CT images were available for all patients with a median surgery-to-scan interval of 1.9 years (range 0.5–9.7 years). A total of 243 postoperative CT images were available, with a median of 2.0 (range 1–10) scans per patient.

Baseline characteristics of patient’s body composition are presented in Table 2. Longitudinal variation in parameters of body composition, expressed as a ratio compared to values at diagnosis (fold change), are presented in Table 3. With the exception of skeletal muscle radiodensity, all parameters of body composition varied significantly after treatment. On average patients lost 13.0 ± 10.3% of their skeletal muscle, 64.5 ± 29.0% of their visceral adipose tissue and 44.2 ± 28.3% of their SAT following treatment for esophageal cancer.

**Table 2:** Baseline parameters of body composition

	All patients	Males	Females	*P*
SM area (cm^2^)	140.5 ± 33.4	152.8 ± 25.0	101.6 ± 26.4	<0.001
VAT area (D)	152.4 ± 90.5	173.2 ± 85.9	85.7 ± 71.6	<0.001
SAT area (cm^2^)	173.8 ± 78.6	168.4 ± 71.9	191.1 ± 96.7	0.229
TAT area (cm^2^)	326.6 ± 144.2	342.3 ± 139.4	276.8 ± 150.8	0.057
SM radiodensity (HU)	36.2 ± 8.7	36.3 ± 7.7	35.9 ± 11.4	0.888
VAT radiodensity (HU)	−91.2 ± 8.5	−92.4 ± 7.8	−87.1 ± 9.5	0.008
SAT radiodensity (HU)	−94.3 ± 9.1	−94.0 ± 8.9	−95.5 ± 9.8	0.498
SMI (cm^2^/m^2^)	47.1 ± 9.9	49.5 ± 8.3	39.3 ± 10.9	<0.001
Sarcopenia (N=)	62 (63.9%)	47 (63.5%)	15 (65.2%)	0.882
Sarcopenic obesity (N=)	38 (39.2%)	31 (41.9%)	7 (30.4%)	0.326
Visceral obesity (N=)	50 (51.5%)	40 (54.1%)	10 (43.5%)	0.375

Values in parentheses are percentages. SM, skeletal muscle. VAT, visceral adipose tissue. SAT, subcutaneous adipose tissue. TAT, total adipose tissue. HU, Hounsfield unit. SMI, skeletal muscle index.

**Table 3:** Longitudinal variation in parameters of body composition (SM, VAT, SAT, SMI fold change) and proportion of patients classified as either Sarcopenic or viscerally obese

	Diagnosis *N* = 97	Post NA-therapy *N* = 61	Post-op 1 year *N* = 73	Post-op 2 years *N* = 54	Post-op 3 years *N* = 39	Post-op 4 years *N* = 21	Post-op 5 years *N* = 15	*P*
SM area	1.00 ± 0.00	0.97 ± 0.09	0.92 ± 0.10	0.92 ± 0.10	0.92 ± 0.12	0.89 ± 0.13	0.84 ± 0.11	<0.001
VAT area	1.00 ± 0.00	0.98 ± 0.52	0.45 ± 0.43	0.45 ± 0.40	0.47 ± 0.32	0.45 ± 0.39	0.41 ± 0.40	<0.001
SAT area	1.00 ± 0.00	0.97 ± 0.68	0.68 ± 0.39	0.69 ± 0.39	0.77 ± 0.36	0.70 ± 0.39	0.76 ± 0.42	<0.001
SMI	1.00 ± 0.00	0.97 ± 0.09	0.92 ± 0.10	0.92 ± 0.10	0.92 ± 0.12	0.89 ± 0.13	0.84 ± 0.11	<0.001
Sarcopenia	62 (63.9)	41 (67.2)	60 (82.2)	42 (77.8)	36 (92.3)	17 (81.0)	12 (80.0)	0.078
Visceral obesity	51 (52.6)	27 (44.3)	4 (5.5)	4 (7.4)	2 (5.1)	1 (4.8)	3 (20.0)	<0.001

SM, skeletal muscle. VAT, visceral adipose tissue. SAT, subcutaneous adipose tissue. SMI, skeletal muscle index. Values in parentheses are percentages.

At baseline the incidence of sarcopenia was 63.5% in males and 65.2% in females (*P* = 0.882). Although rates of sarcopenia tended to be higher with increasing time interval from surgery, this relationship was not statistically significant (*P* = 0.078). In keeping with the observed significant decline in visceral adiposity after surgery (Table 3), rates of visceral obesity fell in the postoperative period.

### Influence of neoadjuvant therapy

Patients who received neo-adjuvant therapy were more likely to report dysphagia (*P* = 0.001) and weight loss (*P* < 0.001) at diagnosis, consistent with their increased cancer stage (*P* < 0.001). One year after surgery patients who received either neoadjuvant chemotherapy (*n* = 46) or chemoradiotherapy (*n* = 20) had an equivalent mean fold change in body composition parameters compared to patients who underwent surgery alone (*n* = 11). At the time of diagnosis, rates of sarcopenia tended to be higher in patients who went on to receive neoadjuvant chemotherapy (69%) and chemoradiotherapy (62%) compared to patients who underwent surgery alone (53%) although this trend was no statistically significant (*P* > 0.05). Rates of visceral obesity were similarly equivalented at diagnosis (52–54%, *P* > 0.05). One year after surgery sarcopenia increased in all treatment groups (surgery alone, 64%; chemotherapy 83%; chemoradiotherapy 80%) although differences between treatment groups were not statistically significant (*P* > 0.05).

### Predictors of body composition variation

The relationship between selected clinical variables and the rate of change in parameters of body composition between diagnosis and 1 year after esophagectomy (Δ cm^2^/year or Δ HU/year) were examined. Compared to females, males had a significantly greater rate of SMI loss (−3.8 ± 4.5 vs. –1.0 ± 2.9 cm^2^/m^2^/year; *P* = 0.015), visceral adipose tissue loss (−87.6 ± 66.4 vs. –33.2 ± 34.5 cm^2^/year; *P* = 0.001) and total adipose tissue loss (−139.6 ± 112.9 vs. –80.8 ± 78.2 cm^2^/year; *P* = 0.001). A significant increase in the rate of change of visceral adipose tissue radiodensity was also observed in males (11.7 ± 8.5 vs. 7.1 ± 7.7 HU/year; *P* = 0.042). Equivalent trends were seen when patients were compared according to histological subtype. This was largely thought to reflect the preponderance of males and females with adenocarcinoma and squamous cell carcinoma, respectively.

Higher ASA grade (II vs. III) was associated with a higher rate of SMI loss (−1.9 ± 3.9 vs. –4.3 ± 4.4 cm^2^/m^2^/year; *P* = 0.011) and visceral adipose tissue loss (−89.0 ± 68.9 vs. –59.7 ± 57.1 cm^2^/year; *P* = 0.049). Patients who reported dysphagia at the time of diagnosis had a higher rate of SAT loss (−58.8 ± 59.0 vs. –29.7 ± 36.0 cm^2^/year; *P* = 0.043). Although neoadjuvant chemoradiotherapy was associated with higher rates of skeletal muscle and adipose tissue loss compared to surgery alone and neoadjuvant chemotherapy, observed differences were not statistically significant. Likewise, weight loss at diagnosis, postoperative complications (Clavien-Dindo grade ≥ III) and clinical stage (I/II vs. III/IV) were not associated with a significant variation in the rate of change in parameters of body composition (*P* > 0.05).

### Patient survival

**Fig. 1:**
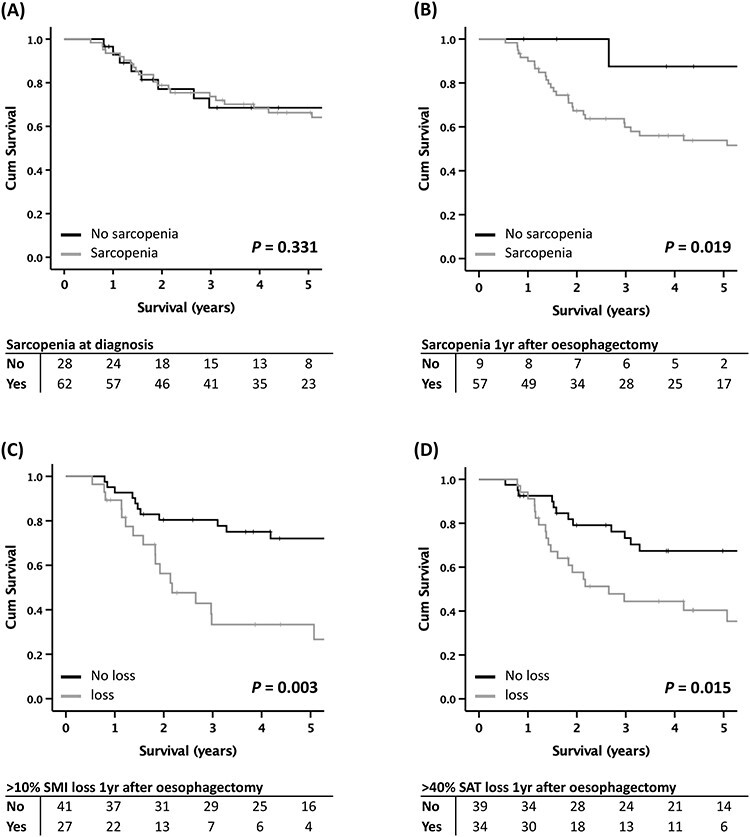
Kaplan–Meier plots comparing overall survival in patients with: (A) sarcopenia at diagnosis; (B) sarcopenia 1 year after esophagectomy; (C) >10% fall of SMI 1 year after esophagectomy and (D) >40% fall of SAT 1 year after esophagectomy.

Median overall survival was 4.4 years (interquartile range 1.8–7.0 years). Sarcopenia at diagnosis was not associated with worse 5-year overall survival (66.3% vs. 68.5%; *P* = 0.331) (Figure 1). Sarcopenia 1 year after esophagectomy was however associated with lower 5-year overall survival (53.8% vs. 87.5%; *P* = 0.019). Survival was also lower in those patients who had >10% decrease in SMI (33.3% vs. 72.1%; *P* = 0.003) and >40% decrease in SAT 1 year after surgery (40.4% vs. 67.4%; *P* = 0.015). Loss (>60%) of visceral adipose tissue was however not associated with worse survival (52.6% vs. 58.8%; *P* = 0.494). Survival did not vary significantly with respect to other clinical factors including, patient age, BMI at diagnosis, ASA grade, tumor histology, clinical and pathological stage. On multivariate analysis a decline in SMI 1 year after surgery was predictive of worse survival (Hazard ratio 0.38, 95% CI 0.20–0.73; *P* = 0.004) (*full details of survival analysis are provided on-line as a supplementary file*).

## DISCUSSION

This is the first study to examine long-term variation in parameters of body composition in patients who have undergone surgery for esophageal cancer. The principal findings of this study were: (i) loss of skeletal muscle at 1 year after esophagectomy was predictive of worse overall survival; (ii) surgery, not neoadjuvant therapy, appeared to be the predominant driver of muscle and fat loss following treatment for esophageal cancer; (iii) a persistent decline in skeletal muscle and adipose tissue after esophagectomy and (iv) significantly higher rates of muscle and adipose tissue loss in males.

Previous studies investigating the impact of parameters of body composition on patient outcomes after treatment for esophageal cancer have typically focused on the assessment of measurements made prior to surgery. These studies have invariably shown that preoperative depletion of skeletal muscle is associated with increased perioperative morbidity and reduced long-term survival.^6^ In a number of studies, assessment of body composition was performed between two time points, typically before and after neoadjuvant therapy. Motoori *et al*. determined that skeletal muscle loss during neoadjuvant chemotherapy, but not preoperative sarcopenia was associated with perioperative complications, including infection.^9^ Similar findings have been reported by other authors.^2^^,^^10^^,^^11^ Recently, Nakashima *et al*. found that patients with a greater reduction in SMI after esophagectomy had worse 5-year overall survival.^12^ Nakashima also identified a 12% decline in SMI within 1 year of esophagectomy that was similar to the findings of the current study. Whilst the predominant histological subtype of esophageal cancer and the ethnic origin of patients were different in these two studies, it is nonetheless encouraging that findings were comparable.

Findings presented herein also suggest that surgery itself has a significant impact on parameters of body composition. This is in keeping with the previous findings of Elliott *et al*. who reported that ongoing muscle loss after esophagectomy was independent of neoadjuvant therapy.^2^ Results confirm that despite significant refinement in surgical techniques and perioperative care, esophagectomy still has the ability to have a lasting impact on patients. Almost all surgeries in the current study were performed via an open approach. It was not therefore possible to determine whether minimally invasive surgical techniques, which are being increasingly employed in the management of esophageal cancer, have a lesser influence the parameters of body composition after esophagectomy.

Low patient numbers, particularly in the surgery only group mean that the influence of neoadjuvant therapy on parameters of body composition remains uncertain. Whilst this study demonstrated a trend towards greater muscle and adipose tissue loss in patients who received neo-adjuvant chemoradiotherapy compared to neoadjuvant chemotherapy or surgery alone, there is insufficient evidence to determine the role of neo-adjuvant as a mechanistic driver of sarcopenia and associated wasting conditions. As well as greater patient numbers, future studies should also control for possible confounding factors such as tumor stage.

This study did not observe an association between major postoperative complications (Clavien-Dindo grade ≥ III) and depletion of parameters of body composition. The long-term effects of esophagectomy on diet, intestinal function and metabolism are also predicted to play a significant, although as yet incompletely understood, role in muscle and adipose tissue loss.

Although previous studies have for the most part focused on skeletal muscle as the primary marker of body composition and its impact on clinical outcomes, the current study has highlighted the potential importance of adipose tissues. Findings show that on average patients lost two thirds of their visceral adipose tissue and almost half of their SAT following esophagectomy. The decline in these parameters appeared to occur largely in the first year after surgery. A well-documented association exists between obesity, principally excess visceral fat and esophageal adenocarcinoma that is relevant not only to the development of the disease but also potentially to treatment response and overall outcome.^13–16^ Whilst many patients with esophageal adenocarcinoma are classified as overweight or obese at the time of diagnosis, disease progression and treatment results in significant loss of this excess adipose tissue. The current study found that on univariate analysis loss of subcutaneous but not visceral adipose tissue was associated with worse overall survival. Despite this interesting and promising finding, further work is needed to better understand the mechanisms and effects of adipose tissue loss during treatment of esophageal cancer.

This study suffers from a number of important limitations. In many cases, it was not possible to acquire all pre- and post-operative CT scans, particularly where imaging had been performed at other institutions. Whilst each center had in place an established schedule for CT imaging before and after esophagectomy, it was common for patients either to depart from this schedule or to become lost to follow-up. The fact that the indication for performing postoperative CT imaging was not reported is also an acknowledged limitation of this study. Selection bias and missing data may therefore have affected findings. The results of this study are largely reflective of outcomes in a Western male population suffering from esophageal adenocarcinoma. It remains therefore unclear whether findings presented herein will be the same for females, patients with squamous cell carcinoma and patients from different racial backgrounds. This study did not assess the potential influence of pre- or peri- operative enteric nutritional supplementation on postoperative body composition. Future studies may benefit from the inclusion of patients receiving non-surgical treatment for esophageal cancer with both curative and palliative intent. Finally, this study did not seek to link changes in body composition to patient reported outcomes relevant to their health-related quality of life. The association between body composition and outcomes relevant to survivorship and quality of life is an important topic and should be the focus of further investigation.

In conclusion, this study provides new insight into the long-term variation in skeletal muscle and adipose tissue in patients undergoing esophagectomy for esophageal cancer. Findings provide further evidence of the importance of body composition, in particular the depletion of skeletal muscle, in predicting survival following esophagectomy. Standardized assessment of body composition may therefore have a future role in supporting prognostication and clinical decision making in esophageal cancer patients.

## Contribution

PRB, GH and DEL designed research, PRB, FK, WJ, FP and KM conducted research, PRB, FK and FP analyzed data or performed statistical analysis, PRB and DEL wrote paper

DEL had primary responsibility for final content. All authors read and approved the final manuscript.

## Supplementary Material

Supplementary_data_file_R1_doab016
