# Supplementary material for: Long-term variation in skeletal muscle and adiposity in patients undergoing esophagectomy
Source: Dis Esophagus. 2021 Apr 5;34(11):doab016. doi: 10.1093/dote/doab016 (PMC8597909; doi:10.1093/dote/doab016)
Supplement: Supplementary_data_file_R1_doab016 [file supplementary_data_file_r1_doab016.docx]

**Supplementary data file**

**Long-term variation in skeletal muscle and adiposity in patients undergoing esophagectomy**

**Authors**

Piers R Boshier^1,2^, Fredrik Klevebro^2^, Wesley Jenq^2^, Francesco Puccetti^2^, Keerthini Muthuswamy^1^, George B Hanna^1^, Donald E Low^2^

**Affiliations**

^1^Department of Surgery and Cancer, Imperial College London, UK

^2^Department of Thoracic Surgery and Thoracic Oncology, Virginia Mason Medical Center, United States of America

**S1.** Results of multivariate analysis of factors predicative of overall survival

|  | **Univariate** | | | **Multivariate** | |
| --- | --- | --- | --- | --- | --- |
|  | **Alive**  **n=49** | **Died**  **n=48** | ***P*** | ***P*** | **HR (95% CI)** |
| **Age (years)** | 62.3 ± 10.6 | 64.1 ± 11.0 | 0.414 | 0.081 |  |
| **Sex (male)** | 39 (79.6) | 35 (72.9) | 0.440 | 0.904 |  |
| **BMI** | 27.0 ± 3.9 | 26.4 ± 4.4 | 0.482 | 0.170 |  |
| **Dysphagia** | 34 (69.4) | 34 (70.8) | 0.876 | 0.678 |  |
| **ASA (III)** | 21 (42.9) | 23 (47.9) | 0.617 | 0.258 |  |
| **Histology (adenocarcinoma)** | 41 (83.7) | 41 (85.4) | 0.812 | 0. 840 |  |
| **cStage** |  |  |  |  |  |
| I/II | 17 (34.7) | 10 (20.8) | 0.532 | 0.221 |  |
| III/IV | 32 (65.3) | 38 (79.2) |  |  |  |
| **pStage** |  |  |  |  |  |
| I/II | 29 (59.2) | 18 (37.5) | 0.087 | 0.094 |  |
| III/IV | 20 (40.8) | 30 (62.5) |  |  |  |
| **CDC ≥III** | 14 (28.6) | 13 (27.1) | 0.870 | 0.830 |  |
| **SMI (FC at 1yr post-op)** | 0.95 ± 0.10 | 0.91 ± 0.11 | 0.149 | 0.703 |  |
| **VAT (FC at 1yr post-op)** | 0.44 ± 0.30 | 0.50 ± 0.63 | 0.601 | 0.187 |  |
| **SAT (FC at 1yr post-op)** | 0.73 ± 0.40 | 0.65 ± 0.43 | 0.428 | 0.644 |  |
| **SMI (>10% loss 1yr post-op)** | 9 (18.4) | 19 (39.6) | 0.078 | 0.004 | 0.38 (0.20 to 0.73) |
| **VAT (>60% loss 1yr post-op)** | 17 (34.7) | 24 (50.0) | 0.627 | 0.459 |  |
| **SAT (>40% loss 1yr post-op)** | 11 (22.4) | 23 (47.9) | 0.081 | 0.233 |  |
| **Sarcopenia at diagnosis** | 28 (57.1) | 34 (70.8) | 0.070 | 0.081 |  |
| **Sarcopenia at 1yr post-op** | 23 (46.9) | 37 (77.1) | 0.002 | 0.062 |  |
| HR, hazard ratio. 95% CI, 95% confidence interval. BMI, body mass index. ASA, American Society of Anesthesiologists score. CDC >III, Clavien-Dindo grade ≥III. FC, fold change. SMI, skeletal muscle index. VAT, visceral adipose tissue. SAT, subcutaneous adipose tissue. | | | | | |
